# Nilotinib as an Independent Risk Factor for Stroke by Accelerated Atherosclerosis

**DOI:** 10.7759/cureus.72952

**Published:** 2024-11-03

**Authors:** Shamira Sibal, Ashwini Patankar, Tushar Raut

**Affiliations:** 1 Neurology, Kokilaben Dhirubhai Ambani Hospital and Medical Research Institute, Mumbai, IND

**Keywords:** chronic myeloid leukemia (cml), drug-induced stroke, long term chemotherapy, multifocal intracranial atherosclerosis, nilotinib, vascular adverse effects, young stroke

## Abstract

Nilotinib, a tyrosine kinase inhibitor (TKI) used in patients of chronic myeloid leukemia (CML), has been known to cause atherosclerosis and arterial stenosis as a rare complication of long-term or high-dose therapy. Patients in this group are more likely to have coronary or peripheral artery disease; intracranial involvement is comparatively uncommon. Furthermore, studies on nilotinib-induced ischemia in Indian populations are scarce. Here, we present a case of ischemic stroke in a patient on long-term nilotinib treatment who, prior to treatment, had no risk factors for stroke. He presented with subacute symptoms of ataxia, motor and sensory deficit, and a raised low-density lipoprotein. MRI revealed multifocal arterial stenosis, as well as areas of infarction and hypoperfusion in the left cerebral hemisphere. Nilotinib therapy was immediately stopped; the patient was treated with dual antiplatelets, statins, and physiotherapy, and he had no major focal deficits on discharge. However, this case serves as a good reminder that even for patients considered to be largely safe from cardiovascular adverse events, regular monitoring of cardiovascular parameters is important so that timely preventive action can be initiated if necessary.

## Introduction

Nilotinib is a drug used to treat patients with chronic myeloid leukemia (CML) who test positive for the Philadelphia chromosome. It works as a tyrosine kinase inhibitor (TKI) and is particularly useful for patients with imatinib intolerance or imatinib-resistant BCR-ABL mutants [1].

A notorious pitfall of treatment with nilotinib is the risk of vascular adverse events, largely due to accelerated atherosclerosis [2], making arterial occlusive events a rare but dreaded complication of nilotinib therapy, especially in the cardiac and peripheral vasculature. This has been prominently featured in the long-term five-year Evaluating Nilotinib Efficacy and Safety in Clinical Trials-Newly Diagnosed Patients (ENESTnd) study [3], which demonstrates that because of such risks, nilotinib should be used with great caution in patients already predisposed to them.

An important aspect brought forth by the second ENESTnd study, which had a 10-year follow-up, was that along with baseline covariates, the duration of treatment is an important determinant of cardiovascular events (CVEs). Patients using nilotinib for over five years are at a much higher risk for CVEs [4]. The present case report demonstrates this factor.

It is also important to consider that while heart disease might be the more common presentation of nilotinib-induced adverse events, cerebrovascular risk is also significant, and must not be discounted [4,5]. Accelerated cerebrovascular atherosclerosis is under-reported in patients on nilotinib. Hence, for the first time to the best of our knowledge, we present a case of an Indian-origin patient with CML who suffered an acute ischemic stroke while on nilotinib treatment, despite having no other baseline risk factors for vascular disease or stroke.

## Case presentation

A 49-year-old man of Indian origin presented with right-ataxic hemiparesis and subsequent symptoms of additional motor and sensory deficits, suggestive of an acute stuttering ischemic stroke.

The patient was undergoing treatment for CML. Initially, he was on imatinib; however, within the first few months, he developed imatinib-related cytopenia and a BCR-ABL T315I mutation. Consequently, he was switched to a regimen of 300 mg nilotinib, taken twice daily. He continued this medication for seven and a half years. He had no prior history of any comorbidities for stroke such as hypertension, dyslipidemia, diabetes mellitus, smoking, alcohol use, or family history of strokes. According to the patient's latest reports before the event, his atherosclerotic cardiovascular disease (ASCVD) risk was 3%, and his Framingham general risk was 3.2%.

At the time of presentation, he reported an imbalance and weakness in the right upper and lower limbs for the past three days and blurred vision in the right eye for the past 24 hours. On examination, he had hemiparesis with a power of 3/5, a visual deficit was found to be in the right temporal hemifield, and it was additionally noted that he had dysarthria, right-sided facial paresis, and a hemi-sensory deficit.

On admission, his National Institutes of Health Stroke Scale (NIHSS) was 4. His blood pressure remained consistently 120/60 mmHg. His metabolic workup confirmed normal total and high-density lipoprotein (HDL) cholesterol levels, thyroid function, glycated hemoglobin (HbA1c), blood glucose, and homocysteine levels (Table 1). However, his low-density lipoprotein (LDL) cholesterol, which was previously within normal limits, was found to be 138 mg/dL.

**Table 1:** Metabolic workup of the patient at the time of stroke presentation HbA1c: glycated hemoglobin; HDL: high density lipoprotein; LDL: low density lipoprotein; TG: triglycerides; PT: prothrombin time; aPTT: activated partial thromboplastin time; INR: international normalized ratio

Parameters	Levels	Normal range
HbA1c	4.70%	up to 6.2%
Cholesterol		
Total	198 mg/dL	0-200 mg/dL
LDL	138 mg/dL	0-100 mg/dL
HDL	44.5 mg/dL	40-60 mg/dL
TGs	116 mg/dL	0-150 mg/dL
Homocysteine	10.03 mmol/L	5.4 to 16.2 mmol/L
Platelets	142000 /mcL	150000-300000 /mcl
APTT	26.4 sec	25 +/- 15 sec
PT	11 sec	upto 12 sec
INR	1.07	upto 1.5
Fibrinogen	228.9 mg/dL	180-350 mg/dL
D-dimer	139 ng/mL	45-500 ng/mL

**Figure 1:**
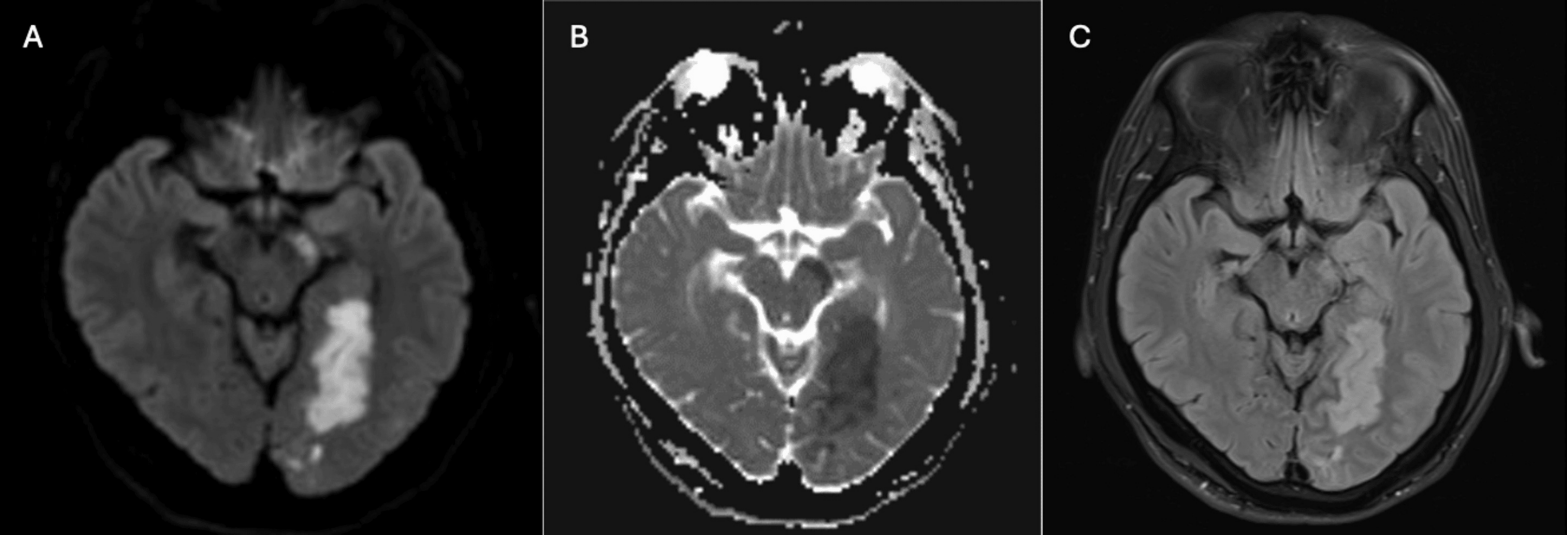
MRI showing areas of brain infarcts Acute left posterior cerebral artery territory infarcts are seen involving the left occipital lobe, left temporal region along para-hippocampal gyrus, left crus of midbrain, and left red nucleus. This is seen in (A) diffusion-weighted imaging, (B) apparent diffusion coefficient,  and (C) fluid-attenuated inversion recovery images.

His MRI brain with angiogram showed acute left posterior cerebral artery (PCA) territory infarcts involving the left occipital lobe, left temporal region along the parahippocampal gyrus, left crus of the midbrain and the left red nucleus; there was hypoperfusion in the left occipitotemporal lobe and middle cerebral artery (MCA) watershed territory (Figure 1).

**Figure 2:**
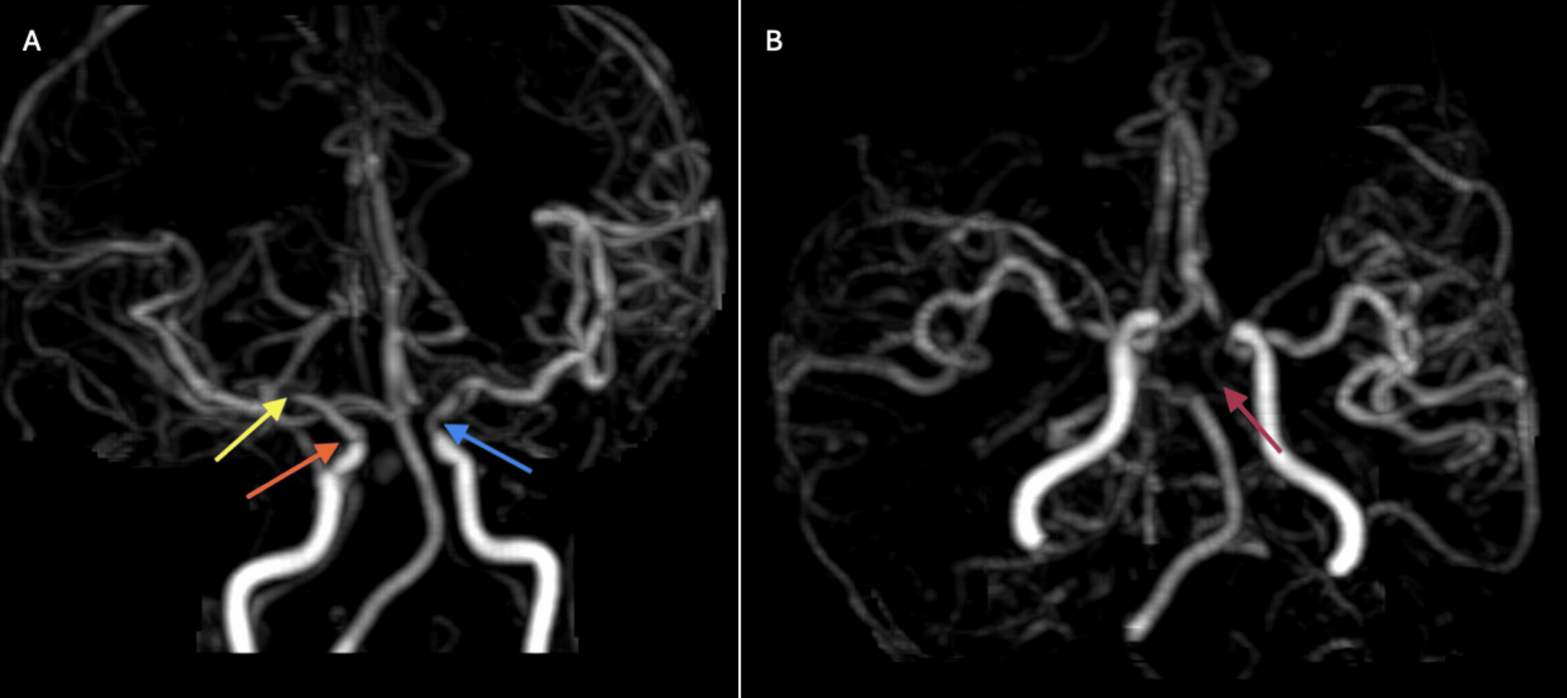
MRA of intracranial arteries (A) Focal high-grade stenosis is seen in the left supraclinoid ICA (blue arrow) causing 70-80% narrowing. The right supraclinoid ICA also showing moderate 40-50% narrowing (orange arrow) and focal moderate narrowing in the proximal M1 segment of right MCA (yellow arrow). (B) Focal occlusion of the P1 segment of left PCA (pink arrow). MRA: magnetic resonance angiography; PCA: posterior cerebral artery; ICA: internal carotid artery; MCA: middle cerebral artery

The angiogram had focal occlusion of the left PCA, P1 segment, and the P2 segment had markedly attenuated flow. There was focal narrowing of the left supraclinoid internal carotid artery (ICA) (70-80%), right supraclinoid ICA (40-50%), right MCA and its M1 segment, and mild narrowing of the left M1 segment of MCA (Figure 2).

The angiogram findings suggested a multifocal intracranial atherosclerotic disease. As the patient did not have any obvious vascular risk factors for stroke, an echocardiogram and electrocardiogram were done to rule out the cardioembolic etiology of stroke. These did not demonstrate any overt cause.

Following the diagnosis of a left PCA ischemic stroke and multi-focal intracranial atherosclerosis, the patient was prescribed dual antiplatelet therapy (150 mg aspirin and 75 mg clopidogrel) along with 40 mg atorvastatin. The metabolic profile revealed elevated cholesterol levels, and the accelerated atherosclerosis was attributed to nilotinib. Consequently, the patient's CML treatment was switched back to imatinib, at a dose of 400 mg once daily.

With physical therapy, the patient experienced symptom recovery and was discharged with an NIHSS score of 2 and a modified Rankin score of 1. On follow-up two months later, his ataxia had resolved, and his muscle strength in the affected limbs remained stable.

## Discussion

Nilotinib is a second-generation TKI used to treat CML. When it comes to nilotinib therapy for patients of CML, the more common adverse effects are hematological abnormalities, rashes, headaches, and serum biochemical derangements such as deranged liver parameters, dyslipidemia, and impaired glucose metabolism. The more worrying complications, such as severe fluid retention and ischemic CVEs, are less frequently seen. 

The incidence of these CVEs is perhaps best illustrated by the five- and 10-year follow-up ENESTnd studies in which the risks of CVEs associated with different doses of nilotinib therapy were studied and compared [3,4]. The CVEs included cardiac ischemic events, ischemic strokes, and peripheral arterial occlusive disease. In the group with 300 mg twice daily dosing of nilotinib, on a median five-year follow-up, 7.5% of patients experienced a CVE, and by 10 years of follow-up on the same dose, approximately 20% of patients treated with nilotinib developed a CVE [4]. Thus, the dose and duration are both found to be contributory to the CVE [4,6]. In our patient, a regimen of nilotinib 300 mg twice daily was ongoing for a period of over seven years, which could explain the ischemic stroke. 

Our patient had no vascular risk factors; however, his MRA of the brain demonstrated intracranial atherosclerotic disease (ICAD), and his metabolic workup demonstrated dyslipidemia which was not present on earlier screening. Nilotinib-induced accelerated atherosclerosis and dyslipidemia, as was seen in our patient, can be explained by various mechanisms that have been studied earlier.

Beyond its suppressive effects on hematopoietic progenitor cells, nilotinib also affects multiple target sites, many of which are thought to contribute to its potential to cause atherosclerosis.

At the vascular cellular level, nilotinib inhibits the TK activity of DDR-1 (an action implicated in plaque formation) and of KIT (which also regulates the mast cell production and release of heparin and tPA) [1]. Additionally, nilotinib stimulates pro-atherogenic cell adhesion and endothelial damage by up-regulating proteins such as endothelial IL-1β (interleukin 1β), ICAM1 (intercellular adhesion molecule 1), VCAM1 (vascular cell adhesion protein 1), and E-selectin, which can recruit pro-inflammatory cells and platelets [7,8]. Other vascular-level nilotinib targets such as KDR and TEK/Tie-2 have also been suggested to cause CVE [2].

With respect to the lipid pathway, nilotinib has been shown to cause a significant increase in the incidence of dyslipidemia by increasing total cholesterol (specifically small dense LDL cholesterol) and LDL cholesterol [9,10]. It has been suggested that this is due to interference with adipocyte lipid accumulation and altered expression of adipogenic regulatory genes (Pparγ, Lpin1, Srebp1) [11]. Insulin resistance and compensatory hyperinsulinemia are also known effects of nilotinib usage, and could also be implicated in dyslipidemia [12].

Nilotinib, on a genetic level, causes insulin resistance modulated by genes such as IRS1 which can accelerate the decomposition of adipose tissue and increase the flow of free fatty acids into the liver. This activates M1-type macrophages which produce chemokines, namely CCL2, IL6, CXCL8, CXCL2, and CXCL20, leading to a complex cascade that encourages atherosclerosis. Factors such as TYROBP and CSF1R also contribute to macrophage-induced lipid dysregulation [13].

Nilotinib also has antiangiogenic effects on endothelial cells, which may impair repair mechanisms related to recanalization and reperfusion after arterial stenosis has occurred [2]. Another proposed mechanism for CVEs induced by the drug is the triggering of vasospasms [14].

Despite its effectiveness, when a patient on nilotinib experiences a CVE, the general consensus is to switch to another TKI [15]. However, prior to starting the drug, a few measures may help in preventing the adverse CVE associated with a potent drug such as nilotinib. These include educating patients about CVE symptoms and routinely monitoring for CVE-related risk factors during follow-ups (see Appendices), as suggested by Manouchehri et al. [8]. If a red flag is identified, secondary prevention with statins, antiplatelet agents, or anticoagulants, as appropriate, should be implemented.

Largely, CVE-related data secondary to nilotinib use has been under-reported in the south-east Asian/Indian population and needs to be monitored.

## Conclusions

CVEs are a well-known adverse effect of nilotinib, but perhaps due to their insidious and chronic nature, patients are not methodically monitored for it. As nilotinib was most likely the cause of multifocal atherosclerosis in our patient, this case serves as a good reminder of nilotinib’s potency as an independent risk factor for CVEs, and that duration of therapy is an important dimension of that risk.

It is therefore advisable for physicians to regularly track a patient’s clinical parameters to pick up on early markers of drug-induced vascular damage, even in patients with no other cardiovascular risk factors, so that preventive measures can be instated as required.

## Appendices

Monitoring parameters with nilotinib 

**Table 2:** Suggested format for monitoring parameters with nilotinib CBC: complete blood counts; LFT: liver function tests; HbA1c: glycated hemoglobin; ECG: electrocardiogram; 2D echo: two-dimensional echocardiography

Parameters	First Visit	1-month follow-up	3-6 month follow-up
Blood Pressure			
Ankle-Brachial Index (ABI)			
CBC / LFT			
Lipid Panel			
HbA1C			
ECG			
2D Echo			
Carotid Doppler			
